# Anti-diabetic activity of crude polysaccharide and rhamnose-enriched polysaccharide from *G. lithophila* on Streptozotocin (STZ)-induced in Wistar rats

**DOI:** 10.1038/s41598-020-57486-w

**Published:** 2020-01-17

**Authors:** Palaniappan Seedevi, Abirami Ramu Ganesan, Meivelu Moovendhan, K. Mohan, Palaniappan Sivasankar, Sivakumar Loganathan, Shanmugam Vairamani, Annaian Shanmugam

**Affiliations:** 1 Centre of Advanced Study in Marine Biology, Faculty of Marine Sciences, Annamalai University, Parangipettai, 608 502 Tamil Nadu India; 2 Department of Environmental Science, Periyar University, Salem, 636011 Tamil Nadu India; 3 Department of Food Science, School of Applied Sciences, College of Engineering, Science and Technology (CEST), Fiji National University, Suva, 5529 Fiji; 4 Bioengineering and Drug Design Laboratory, Department of Biotechnology, Bhupat and Jyoti Mehta School of Biosciences, Indian Institute of Technology, Madras, (IIT-M), Chennai, Tamil Nadu 600036 India; 5 Department of Zoology, Sri Vasavi College, Erode, Tamilnadu India

**Keywords:** Biotechnology, Molecular medicine

## Abstract

The aim of the present study was to elucidate the anti-diabetic effects of the crude polysaccharide and rhamnose-enriched polysaccharide derived from *G. lithophila* on streptozotocin (STZ)-induced diabetic Wistar rats. Treatment with crude polysaccharide and rhamnose-enriched polysaccharide showed increases in body weight and pancreatic insulin levels and a decrease in blood glucose levels compared with control diabetic rats. The blood concentrations of total cholesterol (TC), triglycerides (TGs), low-density lipoprotein (LDL) and very-low-density lipoprotein (VLDL) decreased, and high-density lipoprotein (HDL) increased both in the crude polysaccharide- and rhamnose-enriched polysaccharide-treated rats. Superoxide dismutase (SOD) and glutathione peroxidase (GPx) levels increased, and malondialdehyde (MDA) levels decreased in the livers, kidneys and pancreases of crude polysaccharide- and rhamnose-enriched polysaccharide-treated rats. Immunohistological examination further confirmed that restoration of the normal cellular size of the islets of Langerhans and the rebirth of β-cells were found to be greater in the body region than in the head and tail regions of the pancreas. The crude polysaccharide- and rhamnose-enriched polysaccharide**-**treated diabetic rats showed normal blood glucose levels and insulin production, and reversed cholesterol levels and enzymatic actions. Therefore, rhamnose-enriched polysaccharide from *G. lithophila* acts as a potent anti-diabetic agent to treat diabetes and can lead to the development of an alternative medicine for diabetes in the future.

## Introduction

Diabetes and other associated diseases are major health problems in modern society. Both type 1 and type 2 diabetes comprise abnormalities of insulin action, which includes insulin insensitivity and resistance^1^. Diabetes mellitus (DM) is a group of heterogeneous, hormonal and metabolic disorders characterized by hyperglycaemia and glucosuria, with disturbances in carbohydrate, fat and protein metabolism resulting from defects in insulin secretion, insulin action, or both^2^. Diabetes mellitus is associated with long-term complications such as retinopathy, nephropathy, neuropathy and angiopathy. Furthermore, DM is considered a major risk factor for cardiovascular disorders, namely, ischaemic heart disease, cerebral stroke and peripheral artery disease, leading to increased mortality of patients with diabetes^3^.

In recent times, the most challenging diseases faced by health care professionals are diabetes and other associated disorders. Their increasing prevalence adds a large burden to society and the public health sector^4^. Globally, approximately 171 million people have been affected by diabetes since 2000^5^. The estimated incidence of DM for the year 2010, as given by the International Diabetes Federation, was 239 million^6^. Another study showed that 285 million people worldwide have suffered from diabetes since 2010, among whom ~70% are from lower- and middle-income group countries^7,8^. Therefore, this prediction shows that more than 300 million people will be affected by DM in 2025, which leads to the need forbetter treatment for all diabetes-related chronic disorders^9^. Another study reported that diabetes might affect approximately 366 million people worldwide in the year 2030^5^.

Some disorders of diabetes are characterized by chronic hyperglycaemia and abnormalities in lipid profiles, such as cholesterol, low- and high-density lipoproteins and triglycerides, leading to a series of secondary complications^10,11^. These complications include polyuria, polyphagia, polydipsia, ketosis, retinopathy and cardiovascular disorders^12^. Therefore, several hypoglycaemic agents have been used for the treatment of diabetes mellitus but show serious adverse side effects, such as liver problems, lactic acidosis and diarrhoea^13^. There are several oral anti-diabetic drugs available in the market with varying mechanisms of action for the stimulation of insulin. However, naturally occurring plant-based polysaccharides possess various biological activities, such as antioxidant^14^, antibacterial^15^, anti-diabetic and anticancerous properties. Polysaccharides from *Ophiopogon japonicus* have been reported to exhibit a variety of biological activities, including immuno-stimulation, anti-ischaemia, inhibition of platelet aggregation, hypoglycaemia, etc.^16,17^.

Furthermore, there have been studies that have reported the hypoglycaemic properties of natural products, but the structural elucidation and function of polysaccharides are scarce in the literature. A study conducted on unpurified crude *O. japonicas* polysaccharides showed a significant antagonistic effect on experimental hyperglycaemia in alloxan-induced diabetic mice^16^. However, a purified polysaccharide was found to reduce blood glucose levels and increase insulin levels in streptozotocin-induced diabetic rats, which indicates their potential anti-diabetic nature^16^. Furthermore, there are fewer reports on natural drugs for treating diabetes and related disorders. On the other hand, a balanced diet and regular exercise would be helpful to treat the consequences of diabetes. In critical and necessary situations, the intake of hypoglycaemic medication solves complications related to diabetes^18^. However, these medications are not completely curative and are associated with detrimental effects.

Therefore, natural bioactive compounds for diabetes have attained a greater importance in recent times^19^. To overcome all of these challenges, a search for more effective natural ingredients from sustainable sources, such as seaweed, was found to be attractive by the scientific community. Furthermore, this seaweed is cost-effective, affordable and has no adverse effects even after long-term consumption, which continues to be an important area of research. Hence, the aim of the present study was to determine the anti-diabetic effects of the crude polysaccharide and the rhamnose-enriched polysaccharide derived from *G. lithophila*and to identify the efficiency of these polysaccharides in diabetic-associated disorders in an animal model.

## Methods

### Isolation crude polysaccharide and rhamnose-enriched polysaccharide

The crude polysaccharide and rhamnose-enriched polysaccharide were derived from *G. lithophila* as described by Seedevi^20^.

### Drug and chemicals

Streptozotocin, glutathione peroxidase (GPx), superoxide dismutase (SOD) and malondialdehyde (MDA) were purchased from Sigma Chemicals (USA). The xiaoke pill was obtained from Zhongyi Medicine Limited Company, Guangzhou, Guangdong Province, China. All the other chemicals used in this experiment were of analytical grade.

### Experimental animals

Male albino Wistar strain rats (140–160 g) were obtained from the Central animal house, Rajah Muthiah Institute of Health Sciences, Annamalai University, Tamil Nadu, India. They were housed in polypropylene cages (47 × 34 × 20 cm) lined with husk, refilled at a regular interval of 24 h and kept under a 12-h light, 12-h dark cycle at approximately 22 ± 2 °C. Food and drinking water available *ad libitum*. The rats were fed a standard pellet diet (Parnav Agro Industries Ltd., Maharashtra, India). The pellet diet consisted of 22.02% crude protein, 4.52% crude oil, 3.25% crude fibre, 7.5% ash, 1.38% sand silica, 0.8% calcium, 0.6% phosphorus, 2.46% glucose, 1.8% vitamins and 56.17% nitrogen free extract (carbohydrates). The diet provided metabolisable energy of 3000 Kcal. The experiment was carried out according to the guidelines of the Committee for the Purpose of Control and Supervision of Experiments on Animals (CPCSEA), New Delhi, India and approved by the Animal Ethical Committee of Annamalai University (No. 343; dated 7.9.12)

### Acute toxicity studies

Crude polysaccharide and rhamnose-enriched polysaccharide extracted from *G. lithophila* were studied for their acute toxicity per OECD^21^ guidelines. All healthy Wistar rats in the range of 150–180 g were used in this study. The dosage levels were 0.5, 1, 1.5, 2 and 2 g/kg (p.o.) given to five groups with 5 rats in each group. The mortality and motor behaviour of the treated groups were monitored for 14 days.

### Induction of diabetes in the experimental rats

Diabetes was induced in rats by a single intraperitoneal (i.p.) injection of STZ(55 mg/kg b.wt.) dissolved in freshly prepared 0.01 M citrate buffer, pH 4.5^22^. Three days after STZ treatment, all the experimental rats were measured for their blood glucose levels, with a range of ≥250 mg/dL considered diabetic. Treatments were started on day 4 after STZ injection, and this was considered day 1 for treatment and continued for 30 days.

### Experimental design

The rats were divided into 5 groups (I-V), and each group contained six rats (n = 6).

**Group I:** Normal control rats: Rats were allowed to have free access to a standard pellet diet for 30 days.

**Group II:** Diabetic control rats: The diabetic rats were allowed to have free access to a standard pellet diet for 30 days.

**Group III:** Xiaoke pill-treated diabetic rats (positive control): The diabetic rats were treated with standard pellet diet and xiaoke pill (1.5 g/kg/day, p.o.) for 30 days.

**Group IV:** Crude polysaccharide-treated diabetic rats: The diabetic rats were treated with standard pellet diet and crude polysaccharide (100 mg/kg/day, p.o.) for 30 days.

**Group V:** Rhamnose-enriched polysaccharide-treated diabetic rats: The diabetic rats were treated with a standard pellet diet and rhamnose-enriched polysaccharide (100 mg/kg/day, p.o.) for 30 days.

### Experimental procedure

At the end of the study, all rats were deprived of food overnight and anaesthetized with ketamine (55 mg/kg) through intramuscular injection and then sacrificed. Blood samples were kept in sterile tubes without an anti-coagulant to separate the serum by centrifugation at 3000 rpm for 10 min at room temperature. The samples were preserved at 70 °C until analysis on the total cholesterol (TC), triglycerides (TGs), low-density lipoprotein (LDL), very low-density lipoprotein (VLDL), and high-density lipoprotein (HDL). The organs, such as the liver, kidney and pancreas, were removed and washed in ice-cold normal saline, followed by homogenization with nine volumes of ice-cold 0.9% saline solution in a motor-driven Teflon glass homogenizer to obtain a yield of 10% (w/v) homogenate. The homogenates were centrifuged at 3500 rpm for 10 min at 4 °C, and the supernatant was analysed for the contents of superoxide dismutase (SOD), glutathione peroxidase (GPx) and malondialdehyde (MDA).

### Body weight changes

Once weekly, the body weights were recorded for all the groups of experimental rats.

### Blood glucose estimation

The fasting blood glucose level was determined in all experimental rats initially to determine the diabetic status and once a week thereafter throughout the study period. Blood was obtained by snipping the tails of the rats with the help of a sharp razor, and blood glucose levels were determined using a glucometer (Ultra One Touch, Johnson and Johnson).

### Blood insulin estimation

Blood insulin was measured using the double-antibody method of Bailey and Ahmed-Sorour^23^.

### Estimation of the biochemical parameters

#### Estimation of triglycerides (TG)

The estimation was performed using a GPO kit (Sigma chemical, Maharastra, India) according to the following procedure^24^. The enzyme reagent (1 ml) was added to 10 µl of the sample. The mixture was shaken well and incubated at 37 °C for 10 min. The OD reading was taken at 546 nm using a spectrophotometer (UV-1800, Shimadzu, Kyoto, Japan).

1 $${\rm{Concentration}}\,{\rm{of}}\,\text{Triglycerides}(\text{mg}/\text{dl})=\frac{{\rm{OD}}\,{\rm{of}}\,{\rm{the}}\,{\rm{sample}}}{{\rm{OD}}\,{\rm{of}}\,{\rm{the}}\,{\rm{standard}}}\times 200$$

#### Estimation of total cholesterol (TC)

The total cholesterol estimation was performed using a GPO kit according to the following procedure^24^. One millilitre of the enzyme reagent was added to 10 µl of sample. The mixture was shaken well and incubated at 47 °C for 5 min. The OD reading was noted at 505 nm using a spectrophotometer (UV-1800, Shimadzu, Kyoto, Japan).

2 $${\rm{Concentration}}\,{\rm{of}}\,{\rm{Cholesterol}}\,(\text{mg}/\text{dl})=\frac{{\rm{OD}}\,{\rm{of}}\,{\rm{the}}\,{\rm{sample}}}{{\rm{OD}}\,{\rm{of}}\,{\rm{the}}\,{\rm{standard}}}\times 200$$

### Estimation of HDL Cholesterol

The estimation was performed using a GPO kit according to the following procedure^24^. One millilitre of enzyme reagent was added to 10 µl of sample. The mixture was shaken well and incubated at 37 °C for 5 min. The OD reading was noted at 505 nm using a spectrophotometer (UV-1800, Shimadzu, Kyoto, Japan).

3 $${\rm{Concentration}}\,{\rm{of}}\,{\rm{HDL}}\,{\rm{Cholesterol}}\,(\text{mg}/\text{dl})=\frac{{\rm{OD}}\,{\rm{of}}\,{\rm{the}}\,{\rm{sample}}}{{\rm{OD}}\,{\rm{of}}\,{\rm{the}}\,{\rm{standard}}}\times 100\times 1.1$$

### Estimation of VLDL and LDL Cholesterol

VLDL and LDL cholesterol levels were calculated using Friedewald’s formula^24^.

4 $${\rm{VLDL}}\,(\text{mg}/\text{dl})=\frac{{\rm{Triglycerides}}}{5}$$

5 $${\rm{LDL}}={\rm{Total}}\,{\rm{cholesterol}}\,-\,({\rm{HDL}} \mbox{-} {\rm{CH}}\,+{\rm{VLDL}} \mbox{-} {\rm{CH}})$$

### Estimation of antioxidant markers

#### Estimation of superoxide dismutase (SOD)

The activity of SOD in the liver and kidney tissues was determined according to the procedure of Kakkar^25^. Superoxide radicals react with nitroblue tetrazolium (NBT) in the presence of NADH to produce formazan blue. SOD removes the superoxide radicals and inhibits the formation of formazan blue. The intensity of the colour is inversely proportional to the activity of the enzyme. One unit of enzymatic activity is defined as the enzyme reaction that gives 50% inhibition of NBT reduction in one minute under the assay conditions and is expressed as the specific activity in units/mg protein.

#### Estimation of glutathione peroxidase (GPx)

Reduced glutathione (GSH) is catalysed by glutathione peroxidase (GPx) in the presence of hydrogen peroxide. GSH-Px activity was assessed by the analysis of GSH in the enzymatic reaction^26^. One unit of enzymatic activity of the enzyme unit signifies a decrease in GSH deliberation of 1 µmol/L per minute after deduction of the non-enzymic control^27^.

#### Estimation of malondialdehyde (MDA)

Lipid peroxidation was determined by quantifying the MDA concentration, which was measured in a spectrophotometer by the absorbance of a red-coloured development with thiobarbituric acid^28^. The results are expressed in nmol/mL.

### Method for histology

The pancreas was removed soon after the dissection of the experimental rats and promptly fixed in 10% formalin for histological investigation. The formalin-fixed pancreases were minced to 5 mm to randomize the perception zone and inserted in paraffin. Staining was performed over the paraffin areas with haematoxylin and eosin.

Immunohistochemistry was executed according to the method of Kakimoto *et al*.^29^. Briefly, pancreas paraffin areas were deparaffinized. For insulin immunostaining, the sections were incubated at 4 °C with a primary rabbit polyclonal anti-insulin antibody (Elabscience, India) followed by an HRP-conjugated secondary antibody. Stanning was performed on the segments with 3,3′-diaminobenzidine. Then, images of the areas were captured with an Olympus DP73 advanced camera framework and cellSens Software furnished with an Olympus BX51 magnifying instrument.

### Quantification of the beta-cell area in the intact pancreas

As per the procedure of Kakimoto *et al*.^29^. The entire pancreas was cut into serial 5 μm thick sections spaced 150 μm apart. For analysis, every 30^th^ section was stained. Image processing is a reliable and reproducible method of analysing pancreatic beta cells in islet biology and is equivalent to conventional morphometric analysis. This method is cost effective for analysing the immunohistochemical index. As per the procedure given for the quantification of beta cells by Golson *et al*.^30^, a similar method of quantification was used in our study to quantify the accumulation of beta cell mass and size using Fiji/ImageJ with Java Plugin, the open access software developed by the NIH, USA, and the obtained data were interpreted with MATLAB. The detailed steps involved in image processing were adapted from Golson *et al*.^30^. The longitudinal section of rat pancreas with head, body and tail regions was examined in multiple fields of focus to avoid any error in the surface measurement, and the total mass of the beta cells was extracted for the analysis.

### Statistical analysis

All experimental data were subjected to one-way analysis of variance (ANOVA), and Dunnett’s multiple comparison test (GraphPad Software, USA) was also used to determine the difference among means at the level of *p* < *0.05*. The data obtained from the beta cells of the pancreas were evaluated using MATLAB (version R2017a, Mathworks, MA, USA).

### Ethics approval

All procedures performed in this research work were carried out according to the guidelines of the Committee for the Purpose of Control and Supervision of Experiments on Animals (CPCSEA), New Delhi, India and approved by the Animal Ethical Committee of Annamalai University (No.343; dated 7.9.12). There were no human samples involved in this study.

## Results

### Acute toxicity study

Oral administration of the crude polysaccharide and rhamnose-enriched polysaccharide of *G. lithophila* was found to be safe up to the p.o. dose level 2000 mg/kg, which showed no signs of toxicity. Furthermore, doses up to 2000 mg/kg crude and rhamnose-enriched polysaccharides did not show any negative behavioural changes to motor activity. No mortality was observed up to 14 days. Hence, the median lethal dose (LD_50_) of crude polysaccharide and rhamnose-enriched polysaccharide from *G. lithophila* was determined to be more than 2000 mg/kg body weight (Table 1).

**Table 1:** Observations of rats following administration of crude polysaccharide and rhamnose enriched polysaccharide by oral during acute toxicity study.

Group	Doses mg/ml	Rat effects					
		Crude polysaccharide	Rhamnose enriched polysaccharide	Crude polysaccharide	Rhamnose enriched polysaccharide	Crude polysaccharide	Rhamnose enriched polysaccharide
		D/T	D/T	Mortality latency (h)	Mortality latency (h)	Symptoms of toxicity	Symptoms of toxicity
I	Normal	0/6	0/6	—	—	—	—
II	0.5	0/6	0/6	—	—	—	—
III	1.0	0/6	0/6	—	—	—	—
IV	1.5	0/6	0/6	—	—	—	—
V	2.0	0/6	0/6	>72	>48	Sleepy	Sleepy

### Anti-diabetic activity

#### Changes in body weight

In the present study, the initial body weights of all experimental groups showed no obvious difference between the groups. After 30 days of the experiment, the diabetic control (group II) rats expanded less body weight than the normal control (group I) rats (p < 0.01). Both the crude polysaccharide- and rhamnose-enriched polysaccharide**-**treated groups (IV and V) rat body weights significantly increased, (p < 0.01) as did the xiaoke pill-treated group (III) rats compared to the diabetic control rats (group II) (Table 2).

**Table 2:** Effect of oral administration (30 days) of crude polysaccharide and rhamnose enriched polysaccharide on body weight in STZ diabetic rats.

Group	Administration/ Treatment	Body weight (g)/days				
		−7	1	10	20	30
I	Normal	156 ± 8.9^a^	163 ± 12.0^b^	171 ± 22.7^c^	180 ± 17.3^d^	191 ± 22.5^e^
II	Diabetic	152 ± 10.2^a^	157 ± 6.5^b^	153 ± 9.9^c^	146 ± 13.6^d^	135 ± 11.5^e^
III	Xiaoke pill	151 ± 9.7^a^	159 ± 8.6 ^b^	164 ± 13.7^c^	172 ± 15.4^d^	181 ± 19.5^e^
IV	Crude polysaccharide	155 ± 7.3^a^	161 ± 9.1^b^	168 ± 16.3^c^	176 ± 14.0^d^	184 ± 17.9^e^
V	Rhamnose enriched polysaccharide	158 ± 11.5^a^	166 ± 13.3^b^	173 ± 17.2^c^	180 ± 18.4^d^	191 ± 15.9^e^

^a,b,c,d&e^Comparisons were made between Groups I, II, III, IV and V.

#### Changes in the blood glucose levels in diabetic rats after crude polysaccharide and rhamnose-enriched polysaccharide treatment

On the first day of the experiment, the diabetes-induced groups of rats (II, III, IV and V) showed increased blood glucose levels compared to the control rats (group I) (see Table 3). After 30 days of the experiment, diabetic STZ (group II; 427 ± 4.33 mg/dl) rats exhibited increased blood glucose levels compared with normal control rats (group I; 97 ± 0.59 mg/dl). The crude polysaccharide- and rhamnose-enriched polysaccharide**-**treated rats (group IV and V; 124 ± 1.05 and 117 ± 0.94 mg/dl, respectively) showed reduced blood glucose concentrations compared to the diabetic control (group II) rats. Moreover, in the standard drug xiaoke pill-treated (group III; 109 ± 1.83 mg/dl) rats, the blood glucose concentration decreased significantly (p < 0.01) after 30 days of the experiment, which was similar between the crude polysaccharide (group IV; 124 ± 1.05 mg/dl) and rhamnose-enriched polysaccharide (group V; 117 ± 0.94 mg/dl)-treated groups of rats.

**Table 3:** The effect of crude polysaccharide and rhamnose enriched polysaccharide on blood glucose levels in STZ-induced diabetic rats.

Group	Administration/Treatment	Blood Glucose (mg/dl)			
		1st day	10th day	20th day	30th day
I	Normal control	97 ± 0.44^a^	99 ± 0.79^a^	99 ± 0.48^a^	97 ± 0.59^a^
II	Diabetic control	282 ± 1.56^b^	368 ± 6.91^b^	397 ± 6.22^b^	427 ± 4.33^b^
III	Xioke pills	298 ± 1.56^c^	162 ± 8.36^c^	131 ± 2.02^c^	109 ± 1.83^c^
IV	Crude polysaccharide	308 ± 2.34^d^	169 ± 7.34^d^	137 ± 1.98^d^	124 ± 1.05^d^
V	Rhamnose enriched polysaccharide	301 ± 2.72^e^	166 ± 5.43^e^	128 ± 1.63^e^	117 ± 0.94^e^

^a,b,c,d&e^Comparisons were made between Groups I, II, III, IV and V.

#### Changes in the serum insulin levels of diabetic rats after crude polysaccharide and rhamnose-enriched polysaccharide treatment

The serum insulin levels decreased after 30 days of the experiment in the diabetes-induced (group II; 6.43 ± 0.26 µU/ml) rats compared to the control (group I; 16.94 ± 0.42 µU/ml) rats. However, crude polysaccharide- and rhamnose-enriched polysaccharide**-**treated (groups IV and V) rats showed significantly (p < 0.01) increased serum insulin levels (12.01 ± 0.63 and 15.78 ± 0.22 µU/ml, respectively). The standard drug xiaoke pill-treated (group III; 14.11 ± 0.19 µU/ml) rats showed significantly (p < 0.01) increased serum insulin levels after 30 days of the experiment compared to diabetic control rats (group II). Rhamnose-enriched polysaccharide-treated rats showed similar levels of serum insulin compared to that of standard drug xiaoke pill-treated group of rats (Table 4).

**Table 4:** The effect of polysaccharide and rhamnose enriched polysaccharide on insulin levels in STZ-induced diabetic rats.

Group	Administration/Treatment	Serum insulin (µU⁄ml)
I	Normal control	16.94 ± 0.42^a^
II	Diabetic control	6.43 ± 0.26^b^
III	Xioke pills	14.11 ± 0.19^c^
I	Crude polysaccharide	12.01 ± 0.63^d^
V	Rhamnose enriched polysaccharide	13.78 ± 0.22^e^

^a,b,c,d&e^Comparisons were made between Groups I, II, III, IV and V.

#### Changes in the blood fat levels

**Figure 1:**
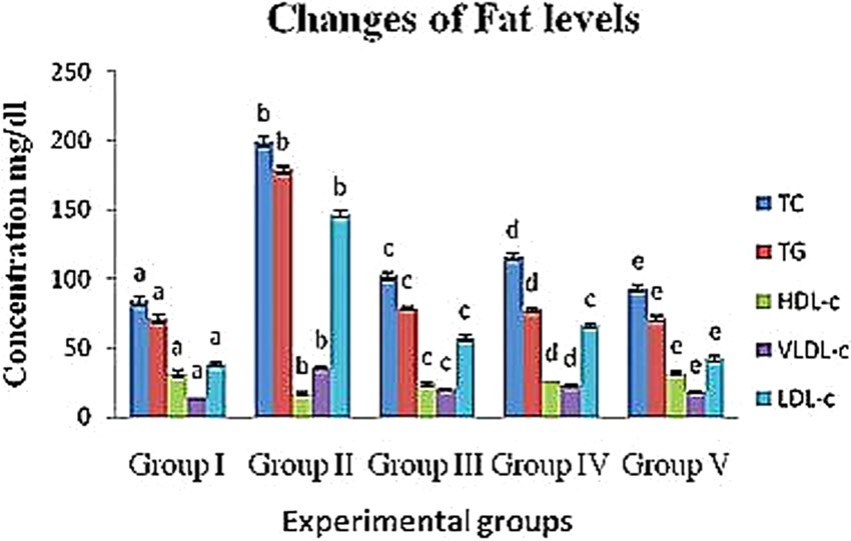
Changes of crude polysaccharide and rhamnose enriched polysaccharide on fat level in STZ diabetic rats. (**TC**) Total cholesterol, (**TG**) Triglycerides, (**HDL**) High density lipoprotein, (**VLDL**) Very low density lipoprotein and (**LDL**) Low density lipoprotein. ^a,b,c,d&e^Comparisons were made between Groups I, II, III, IV and V.

The blood concentrations of triglycerides, total cholesterol, HDL, LDL and VLDL are shown in Table 5 and Fig. 1. In the present study, diabetic control (group II) rats showed high concentrations of total cholesterol, triglycerides, VLDL-cholesterol and LDL-cholesterol in the blood (199.8 ± 3.5, 179.2 ± 2.30, 35.84 ± 0.66 and 146.86 ± 2.32 mg/dl, respectively) and a low concentration of HDL-cholesterol (17.1 ± 0.92 mg/dl) compared to the normal control group (group I) rats at the end of the experiment. Both crude polysaccharide and rhamnose-enriched polysaccharide treatment (groups IV and V) showed significantly decreased levels of TGs, TC, LDL-cholesterol & VLDL-cholesterol compared to control diabetic (group II) rats (116.1 ± 2.38, 77.6 ± 1.45, 23.22 ± 0.86 and 66.48 ± 2.11 mg/dl for group IV and 93.2 ± 2.38, 71.6 ± 1.45, 18.64 ± 0.69 & 42.26 ± 1.6 mg/dl for group V, respectively); however, the HDL-cholesterol levels (26.4 ± 0.65 and 32.3 ± 1.88 mg/dl, respectively) increased and were similar to that of the reference value (group I; 31.62 ± 2.06 mg/dl).

**Table 5:** Effect of crude polysaccharide and rhamnose enriched polysaccharide on fat level in STZ diabetic rats.

Group	Administration/Treatment	Concentration mg/dl				
		Total cholesterol	Triglycerides	HDL- cholesterol	VLDL- cholesterol	LDL- cholesterol m
I	Normal	84.5 ± 3.33^a^	71.5 ± 2.81^a^	31.62 ± 2.06^a^	14.3 ± 0.38^a^	38.58 ± 1.5^a^
II	Diabetic	199.8 ± 3.5^b^	179.2 ± 2.30^b^	17.1 ± 0.92^b^	35.84 ± 0.66^b^	146.86 ± 2.32^b^
III	Xiaoke pill	102.3 ± 2.38^c^	79.2 ± 1.14^c^	24.3 ± 0.11^c^	20.46 ± 0.93^c^	57.54 ± 1.45^c^
IV	Crude polysaccharide	116.1 ± 2.38^d^	77.6 ± 1.45 ^d^	26.4 ± 0.65 ^d^	23.22 ± 0.86 ^d^	66.48 ± 2.11 ^d^
V	Rhamnose enriched polysaccharide	93.2 ± 2.38^e^	71.6 ± 1.45^e^	32.3 ± 1.88^e^	18.64 ± 0.69^e^	42.26 ± 1.6^e^

^a,b,c,d&e^Comparisons were made between Groups I, II, III, IV and V.

#### Change of SOD levels in diabetic rats

**Figure 2:**
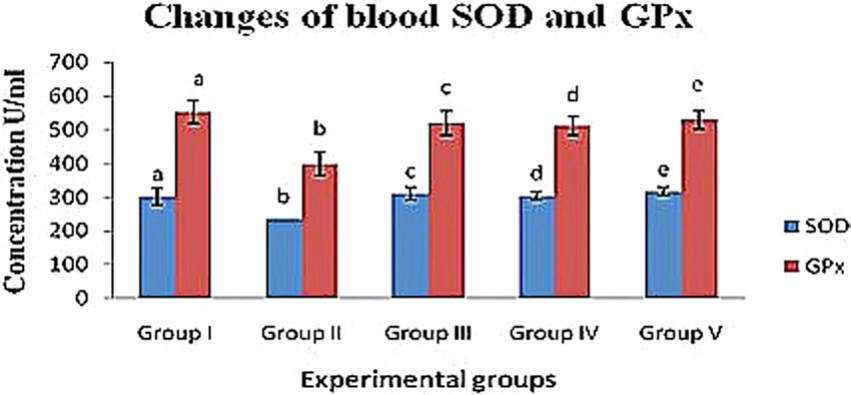
Effects of crude polysaccharide and rhamnose enriched polysaccharide on blood (**GPx** and **SOD**) level in STZ diabetic rats. (**SOD**) Superoxide dismutase and (**GPx**) Glutathione peroxidase. ^a,b,c,d&e^Comparisons were made between Groups I, II, III, IV and V.

**Figure 3:**
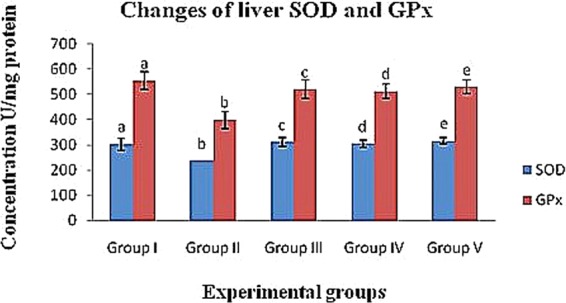
Effects of crude polysaccharide and rhamnose enriched polysaccharide on liver (**GPx** and **SOD**) level in STZ diabetic rats. (**SOD**) Superoxide dismutase and (**GPx**) Glutathione peroxidase. ^a,b,c,d&e^Comparisons were made between Groups I, II, III, IV and V.

**Figure 4:**
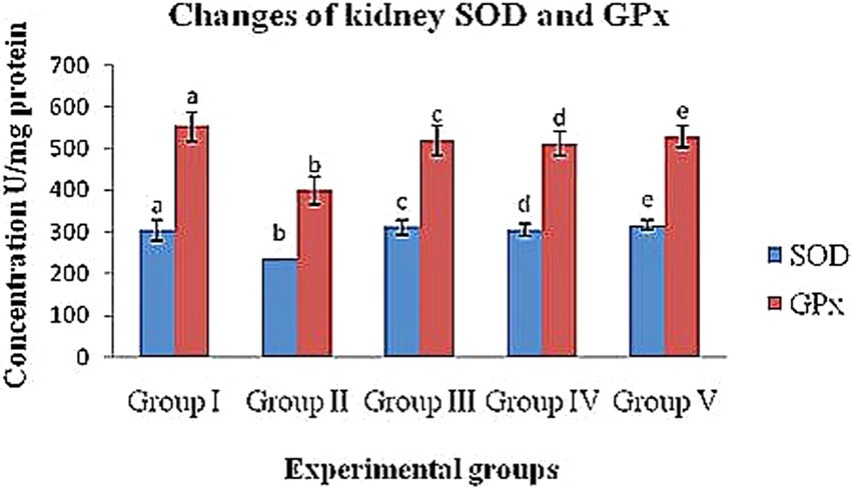
Effects of crude polysaccharide and rhamnose enriched polysaccharide on kidney (**GPx** and **SOD**) level in STZ diabetic rats. (**SOD**) Superoxide dismutase and (**GPx**) Glutathione peroxidase. ^a,b,c,d&e^Comparisons were made between Groups I, II, III, IV and V.

The levels of the enzyme SOD in the experimental diabetic groups increased in the blood, liver and kidney (group II; 202.11 ± 5.89 U/ml, 236.43 ± 31.69 U/mg protein and 254.32 ± 20.62 U/mg protein, respectively) compared to the control (group I) rats (273.01 ± 6.23 U/ml, 302.10 ± 24.56 U/mg protein and 331.34 ± 23.43 U/mg protein, respectively). In contrast, the crude polysaccharide-treated (group IV) rats (236.14 ± 9.33 U/ml, 304.13 ± 13.77 U/mg protein and 291.54 ± 18.58 U/mg protein) and rhamnose-enriched polysaccharide-treated (group V) rats (244.05 ± 9.87 U/ml, 316.22 ± 11.89 U/mg protein and 318.17 ± 22.08 U/mg protein) showed significantly (p < 0.05) decreased SOD levels in the blood, liver and kidney compared to the diabetic control (group II) rats. The xiaoke pill-treated rats (group III) reported SOD levels of 233.06 ± 15.23 U/ml, 298.44 ± 20.47 U/mg protein and 311.09 ± 17.83 U/mg protein in blood, liver and kidney, respectively (Table 6 & Figs. 2–4).

**Table 6:** Effects of crude polysaccharide and rhamnose enriched polysaccharide on blood, liver and kidney (MDA, GPx and SOD) level in STZ diabetic rats.

Sample	Enzymes	Experimental groups				
		I	II	III	IV	V
Blood	SOD (U/ml)	273.01 ± 6.23^a^	202.11 ± 5.89^b^	233.06 ± 15.23^c^	236.14 ± 9.33^d^	244.05 ± 9.87^e^
	GPx (U/ml)	857.21 ± 31.54^a^	512.10 ± 41.76^b^	809.15 ± 33.72^c^	853.19 ± 23.45^d^	864.28 ± 21.98^e^
	MDA (nmol/ml)	8.11 ± 1.21^a^	13.81 ± 1.43^b^	8.16 ± 1.66^c^	9.17 ± 1.42^d^	8.91 ± 1.01^e^
Liver	SOD (U/mg protein)	302.10 ± 24.56^a^	236.43 ± 31. 69^b^	311.09 ± 17.83^c^	304.13 ± 13.77^d^	316.22 ± 11.89^e^
	GPx (U/mg protein)	553.18 ± 35.34^a^	398.56 ± 33.54^b^	520.29 ± 36.57^c^	511.26 ± 28.98^d^	529.29 ± 27.59^e^
	MDA (nmol/mg protein)	1.29 ± 0.11^a^	2.99 ± 0.44^b^	1.99 ± 0.25^c^	1.56 ± 0.34^d^	1.39 ± 0.22^e^
Kidney	SOD (U/mg protein)	331.34 ± 23.43^a^	311.09 ± 17.83^b^	298.44 ± 20.47^c^	291.54 ± 18.58^d^	318.17 ± 22.08^e^
	GPx (U/mg protein)	327.51 ± 14.05^a^	217.69 ± 17.23^b^	298.37 ± 9.11^c^	282.40 ± 10.46^d^	307.19 ± 7.19^e^
	MDA (nmol/mg protein)	2.21 ± 0.31^a^	3.81 ± 0.38^b^	2.61 ± 0.19^c^	2.37 ± 0.23^d^	2.31 ± 0.17^e^

^a,b,c,d&e^comparisons were made between Groups I, II, III, IV and V.

#### Changes in GPx levels of diabetic rats

The GPx levels of the diabetic control (group II) rats was reduced in the blood, liver and kidney (512.10 ± 41.76 U/ml, 398.56 ± 33.54 U/mg protein and 217.69 ± 17.23 U/mg protein) compared to that of the control rats (group I; 857.21 ± 31.54 U/ml, 553.18 ± 35.34 U/mg protein and 327.51 ± 14.05 U/mg protein, respectively). Crude polysaccharide-treated rats (group IV; 853.19 ± 23.45 U/ml, 511.26 ± 28.98 U/mg protein and 282.40 ± 10.46 U/mg protein) and rhamnose-enriched polysaccharide-treated (group V) rats (864.28 ± 21.98 U/ml, 529.29 ± 27.59 U/mg protein and 307.19 ± 7.19 U/mg protein) recorded significantly (p < 0.05) increased levels of GPx in the blood, liver and kidney compared to the diabetic control (group II) rats. The xiaoke pill-treated rats (group III) recorded significantly increased (809.15 ± 33.72 U/ml, 520.29 ± 36.57 U/mg protein and 298.37 ± 9.11 U/mg protein) levels of GPx in the blood, liver and kidney compared to the diabetic control (group II) rats (Table 6 & Figs. 2–4).

#### Changes in MDA levels in diabetic rats

**Figure 5:**
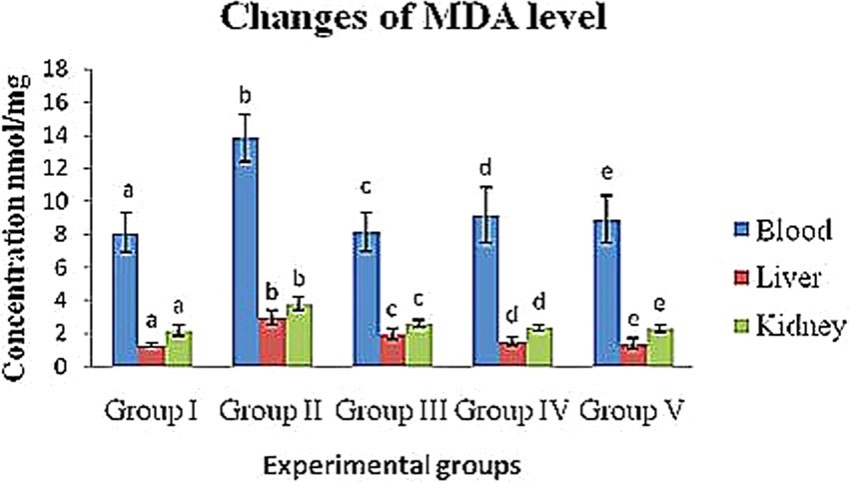
Changes of crude polysaccharide and rhamnose enriched polysaccharide on blood, liver and kidney (MDA) level in STZ diabetic rats. (MDA) Malondialdehyde. ^a,b,c,d&e^Comparisons were made between Groups I, II, III, IV and V.

The MDA levels of the diabetic control (group II) rats increased in the blood, liver and kidney (13.81 ± 1.43, 2.99 ± 0.44 and 3.81 ± 0.38 nmol/ml, respectively) compared to that of the control (group I) rats (8.11 ± 1.21, 1.29 ± 0.11 and 2.21 ± 0.31 nmol/ml, respectively). However, crude polysaccharide-treated (group IV) rats (9.17 ± 1.42, 1.56 ± 0.34 and 2.37 ± 0.23 nmol/ml) and rhamnose-enriched polysaccharide-treated (group V) rats (8.91 ± 1.01, 1.39 ± 0.22 and 2.31 ± 0.17 nmol/ml) showed a significant (P < 0.05) decrease in MDA levels in the blood, liver and kidney compared to the diabetic control (group II) rats. Xiaoke pill-treated rats (group III) reported significantly reduced (8.16 ± 1.66, 1.56 ± 0.34 and 2.37 ± 0.23 nmol/ml) levels in the blood, liver and kidney compared to the diabetic control (group II) rats (Table 6 & Fig. 5).

#### Changes in the histopathology of the pancreas

**Figure 6:**
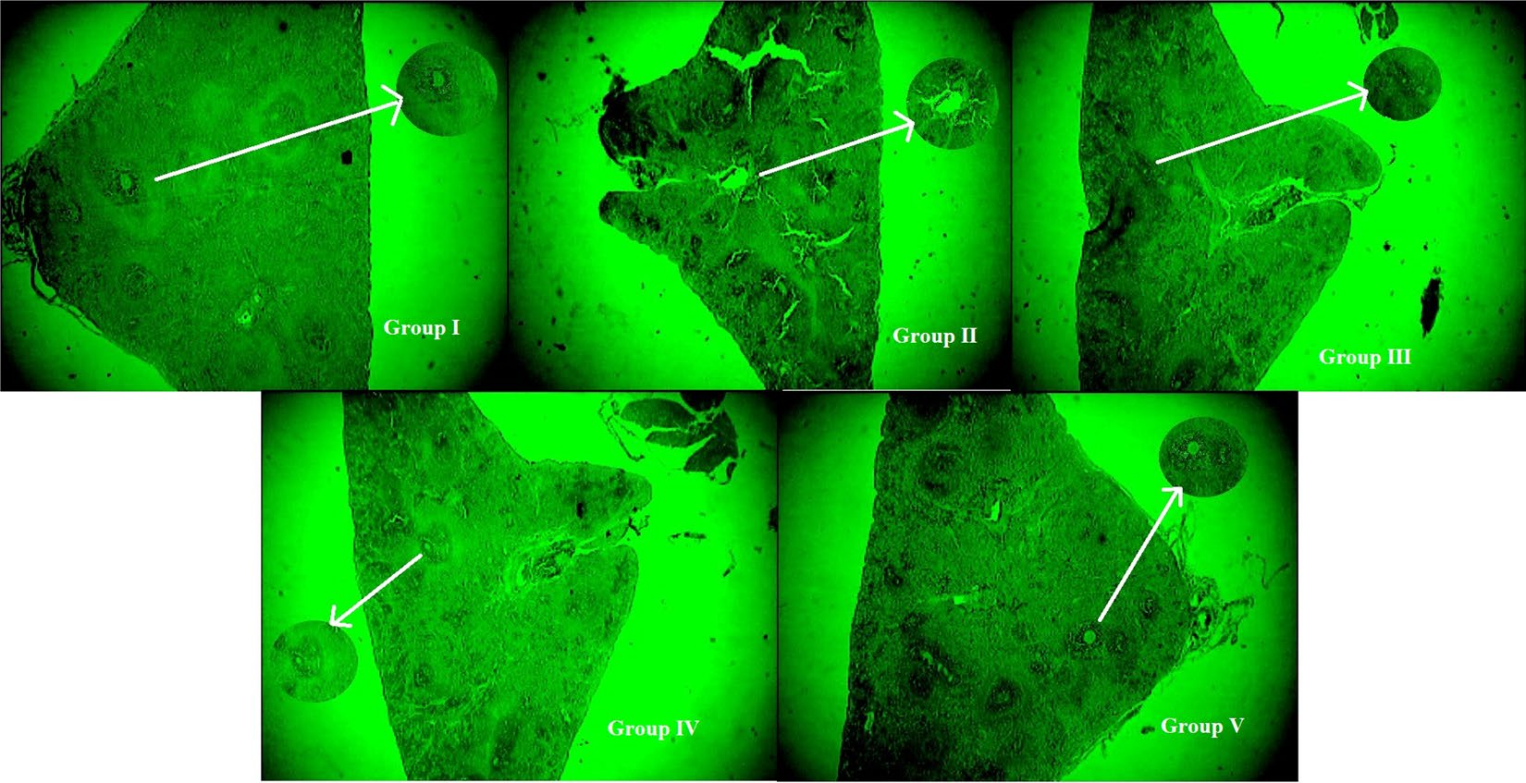
Histopathological changes and beta cells of pancreas in rat administrated with crude polysaccharide and rhamnose enriched polysaccharide from *G. lithophila*. Group-I, Normal (**control**) pancreas showing no pathological changes with; (**a**) normal islets of Langerhans with (**b)** β-cells; Group-II, (**a**) Pancreas (**diabetic control**) showing moderate to marked diffused necrotic changes as a result of which there was significant reduction in the number of (**b**) β-cells in the islets of Langerhans; Group-III (**positive control**), showed slight development of (**a**) islets of Langerhans along with (**b)** β-cells; Group-IV, (**a**) showed improvement of islets of Langerhans and (**b**) specify the improvement of β-cells; Group-V, (**a**) clearly indicated the restoration of normal cellular size of islets of Langerhans and (**b**) designated to rebirth of β-cells.

The control rats (group I) showed no pathological changes in the pancreatic cellular architecture. The diabetic control (group II) rats showed moderate to marked diffused necrotic changes in the pancreas as a result of the significant reduction in the number of β-cells in the islets of Langerhans. The positive control, the xiaoke pill-treated (group III) rats, showed a slight development of islets of Langerhans along with β-cells. The crude polysaccharide-treated rats (group IV) showed an improvement in islets of Langerhans and specific improvement in the β-cells, and rhamnose-enriched polysaccharide-treated (group V) rats showed significant regeneration of β-cells when compared to the diabetic control (Fig. 6).

### Quantification of beta cells

To measure the *in situ* beta cell zone of the rat pancreas for all experimental groups of rats, an entire pancreas was cut as a virtual cut with high magnification to evaluate the whole dissemination of beta cells *in situ* for analysis. Macro-plugin was used in Fiji/ImageJ for the size distribution from the pancreatic images. The images with beta cells were segmented for the surface measurement, total area, circularity and Feret’s diameter and then evaluated. The images were physically checked the region of interest (ROI) produced via the programmed threshold was revised to ensure ROI displays of only the beta cells region. Then, the examinations were undertaken, and each islet area was traced manually to calculate the surface measurement.

**Figure 7:**
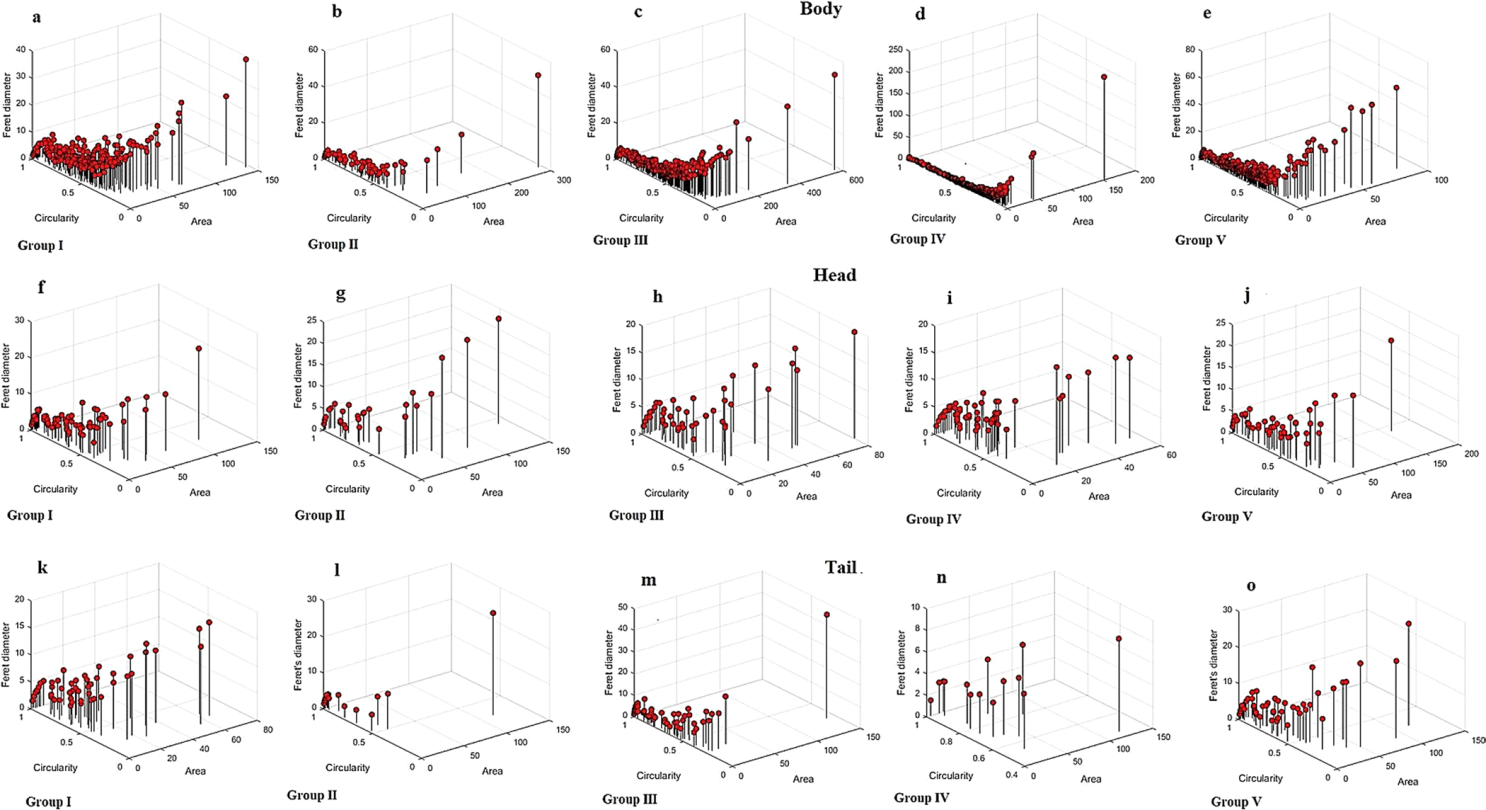
Quantitative analysis of individual beta cell distribution in the rat pancreas administrated with crude polysaccharide and rhamnose enriched polysaccharide from *G. lithophila*. 3D visualization in scatter plot of islet size distribution with beta cells in various size and shape. Circularity represents the roundness of structure (1.0 denotes perfect circle), Feret’s diameter (µm) displays the distance within structure. (**a**–**e**) showed the quantification of beta cells in body region of pancreas from group I–V, (**f**–**j**) showed the quantification of beta cells in head region of pancreas from group I–V, (**k**–**o**)showed the quantification of beta cells in tail region of pancreas from group I–V.

Dynamic changes of cell arrangement throughout the islet measurements were observed for each experimental group. The immunostaining image of the rat pancreas and proliferation of the beta cells in the experimental group are shown in Fig. 7a–o. As per Olehink *et al*.^19^ 60% of beta cells in the total endocrine surface are <100 μm in diameter. Similarly, most of the islet areas covered <100 to 150 μm, as shown in Fig. 7a–e (group I) shows the exception, where the healthy pancreas beta cells are distributed for up to 600 μm of the pancreatic body region. Three-dimensional scatter plots show the islet area, circular shape and Feret’s diameter. There were significant changes found in group III (positive control), with the accumulation of beta cells found in the body region (Fig. 7a–e) compared with the head (Fig. 7f–j) and tail regions (Fig. 7k–o) of the pancreas. Groups IV and V also showed high proliferation rates but were not equivalent to group III. There were few beta cells observed in the STZ-treated rat pancreas, i.e., group II, compared to all the diabetic rats. The control group (group I) rat pancreas consisted of a high number of beta cells with intact islet areas. Overall, the distribution of beta cells from the islet mass shows that the greater accumulation in the body region gradually decreases to the tail region, although there is some fluctuation.

## Discussion

Streptozotocin is one of the most commonly used substances to induce diabetes in rats. This toxin causes the death of pancreatic cells by alkylation of DNA, resulting in reduced DNA synthesis and release of insulin^29^. STZ administration of more than 40 mg/kg to rats is capable of destroying pancreatic β-cells and causing the development of constant hyperglycaemia, a situation parallel to diabetes in humans^29,30^. In the present study, administration of STZ at 55 mg/kg to rats was found to be capable of increasing blood glucose levels on the 3^rd^ day of the experimental period, which may be due to the destruction of pancreatic islets and the death of β-cells. In diabetes mellitus, body cells are unable to utilize glucose as a source of energy and proteins are instead used as energy sources. This leads to a decrease in protein storage and reduces body weight^31^. In the present study, streptozotocin-induced diabetic rats showed a decrease in body weight throughout the experimental period. Oral treatment with crude polysaccharide and rhamnose-enriched polysaccharide from *G. lithophila* to diabetic rats for 30 days resulted in a significant improvement in body weight compared to the control rats.

Diabetic mellitus is mainly associated with multiple metabolic disorders, amongst which lipid metabolism is relatively affected by high concentrations of triglycerides and total cholesterol and low levels of HDL cholesterol, as found in many cases^32^. The concentrations of TC, TGs and LDL cholesterol rise, and HDL cholesterol levels decrease in the blood, contributing to secondary complications of diabetes^32^. In the present study, the lipid profile of diabetic rats changed to show increased levels of TC, TGs, LDL cholesterol and VLDL cholesterol and decreased HDL cholesterol levels. Treatment with crude polysaccharide and rhamnose-enriched polysaccharide resulted in an elevated lipid profile, such as total cholesterol (TC), triglycerides (TGs) and LDL-cholesterol, similar to the reference level, and significantly increased the concentration of HDL-cholesterol. This finding was desirable in this study. These results may be associated with the possible protective effect of the polysaccharides to pancreatic β-cells. In addition, these effects may be due to the low activity of cholesterol biosynthetic enzymes or the insignificant level of lipolysis under the control of insulin^33^. Therefore, glycol metabolism and lipid metabolism may improve the renovation of β-cells and increase insulin secretion^34^.

In diabetes, hyperglycaemia results in the generation of free radicals, which weakens the body’s defence mechanisms, thus leading to the disruption of cellular functions, oxidative damage to membranes and an enhanced susceptibility to lipid peroxidation^35^. Increased glycated Cu- and Zn-SOD activity have been reported in severe diabetes^36^. SOD and GPx are important enzymatic antioxidants that could be helpful in preventing lipid peroxidation and are involved in cellular defence mechanisms, thus protecting tissues against oxidative damage^37^. Under hyperglycaemic conditions, antioxidants are thought to regenerate the damaged extracellular matrix proteins and cell growth^35^. In addition, an increase in pancreatic MDA content has been reported previously in hyperglycaemic rats^37^.

Further, additional stimulation of MDA is associated with increased serum glucose levels and the destruction of ROS. Glucose possibly increases the formation of ROS through the non-enzymatic glycation of proteins, as well as through auto-oxidation^38^. The oxidative degradation of fructosamines may also contribute to oxidative stress in hyperglycaemia^36^. In our study, a significant increase in MDA levels and a reduction in SOD and GPx in the serum, livers and kidneys of STZ-induced diabetic rats was seen. However, the treatment of rats with crude polysaccharide and rhamnose-enriched polysaccharide lowered MDA levels and restored SOD and GPx activities to near normal levels. Similar results were observed with *Achyranthes bidentata* polysaccharides, which increased MDA concentrations and reduced other antioxidant enzymes, such as SOD and GPx, in the blood, liver and kidney tissues of STZ-induced diabetic rats^34^.

The enhanced activity of SOD and GPx in the present study may contribute to the potential effect of rhamnose-enriched polysaccharides on the scavenging of oxygen free radicals. Pancreatic β-cells may be protected from the oxidative damage induced by STZ. Therefore, it could be assumed that rhamnose-enriched polysaccharide has a direct protective effect against diabetes by decreasing the oxidative stress and preserving pancreatic β-cell damage, and this may be due to the reaction of rhamnose-enriched polysaccharide, which improves glycometabolism. In the present study, the pancreases of STZ-induced rats showed a significantly reduced size and number of β-cells in the islets of Langerhans after 30 days. Furthermore, the pancreases of rhamnose-enriched polysaccharide-treated rats showed the restoration of normal cell size of the islets of Langerhans and regenerated β-cells.

Chronic hyperglycaemia has deleterious effects on pancreatic β-cells by inducing oxidative stress^39^, which has been found to be more problematic than in other cells because β-cells possess the lowest intrinsic antioxidant defences^40^. In the present study, treatment with crude polysaccharide and rhamnose-enriched polysaccharide from *G. lithophila* to diabetic rats may prevent the development of diabetes by reducing glucose concentration. However, no significant differences were observed between the crude polysaccharide- and rhamnose-enriched polysaccharide-treated groups. The possible reasons may be that crude and rhamnose-enriched polysaccharides increased insulin release through antioxidant activity or the regeneration of β-cells^38^. Pancreatic β-cells are highly specialized cells responsible for producing the insulin required by an organism to maintain glucose homeostasis. Defects in the development, maintenance or expansion of β-cell mass can result in the impairment of glucose metabolism and diabetes^41^.

In the present study, STZ-induced rats showed a reduction in insulin levels in the serum compared to the control group. Treatment with crude polysaccharide and rhamnose-enriched polysaccharide significantly increased insulin levels in STZ rats compared to the diabetic control. The xiaoke pill-treated group rats also showed similar insulin levels that were on par with the rhamnose-enriched polysaccharide-treated group of rats. This strengthens the insulin-mimicking effect of rhamnose-enriched polysaccharides by regenerating insulin-producing cells.

The area of the beta cells was found in a limited number in the negative control (group II) rats with a depleted number of beta cells; all these areas were manually quantified in threshold images for their particle size. Furthermore, manual tracing of these regions was labour-intensive due to the irregularly shaped islets and beta cells, as has been observed in previous studies as well^41^. The present study shows that the expansion in beta cell mass is mainly due to the increase in beta cell replication, irrespective of the progenitors of beta cells that exist in the mature pancreas. In this study, mechanical methods were used for evaluating the islet region and insulin-positive region with beta cells in the insulin immunostained pancreas using Fiji/ImageJ software (Fig. 7). MATLAB simulation has been developed to ideally quantify the islet region from pancreatic areas (head, body and tail). The role of image investigation is to convert the subjective histological observations to quantitative features. The improvement of computerized vision has allowed the entire slide access with high resolution and has allowed for more tissues to be examined. This method of evaluation provides reliable information and is widely used in biomedical image analysis^19,41^. Overall, the distribution of beta cells from the islets of Langerhans shows greater accumulation in the body region and gradually decreases in the tail region. However, other studies conducted in the human pancreas have shown that more beta cells were present in the tail region of diabetes patients^41^.

The insulinogenic effect of various medicinal plant extracts resulting in the activation of β-cells has been reported^42,43^. The possible mechanism through which they exert their anti-hyperglycaemic effects might have be due to the increased release of insulin from β-cells or through the regeneration of β-cells. Under these circumstances, a number of other plants have also been reported to have anti-hyperglycaemic activity with a stimulatory effect on insulin release^37^.

## Conclusions

The present study suggested that rhamnose-enriched polysaccharides from *G. lithophila* could be used as an anti-diabetic agent due to the insulin-mimetic effects to reverse the blood glucose concentration, restore insulin production, improve cholesterolemic and enzymatic action and regenerate β-cells. Therefore, the rhamnose-enriched polysaccharide from *G. lithophila* can act as a potent anti-diabetic agent to treat diabetic patients, which will help future research working on the formulation of an alternative medicine for diabetes.

### Limitations of the study

The effects of rhamnose-enriched polysaccharides in STZ-induced diabetic rats were studied in this work, but the mechanism of action at the molecular level is required to examine their role. A molecular mechanism study is therefore required to determine the improvement of dyslipidaemic conditions in diabetes for further drug discovery. Furthermore, beta cell accumulation was found to be high in the body region of the pancreas in our study. Compared to this result, beta cells originated more in the body and tail region of the human pancreas (Freeby *et al*.^44^); however, Rahier *et al*.^45^ reported a greater accumulation of beta cells in the posterior head region of the human pancreas. Therefore, the role of rhamnose-enriched polysaccharides in the increment of beta cells in the body region of the rat pancreas needs to be studied further.

## Data Availability

All data generated or analyzed during this study are included in this published article.
